# Clinical features and outcomes of germline mutation *BRCA1*-linked versus sporadic ovarian cancer patients

**DOI:** 10.1186/s13053-015-0044-z

**Published:** 2016-01-08

**Authors:** Agnieszka Synowiec, Gabriel Wcisło, Lubomir Bodnar, Bohdan Górski, Jolanta Szenajch, Katarzyna Szarlej-Wcisło, Cezary Szczylik

**Affiliations:** Department of Oncology, Military Institute of Medicine, 128 Szaserow Str., 04-141 Warsaw, Poland; Department of Genetics and Pathology, Pomeranian Medical University, Szczecin, Poland

**Keywords:** Germline mutations of *BRCA1*, Ovarian cancer, Chemotherapy, Survival

## Abstract

**Background:**

The role of germline mutations in *BRCA1* and *BRCA2* genes in the risk of the development of ovarian cancer is clinically well established. *BRCA1/2* testing seems to have increasing role in clinical management in patients with advanced ovarian cancer who require treatment with poly(ADP-ribose) polymerase inhibitors.

**Methods:**

Between 2002 – 2008, 125 consecutive patients with ovarian cancer were categorized as having three founder mutations in the *BRCA1* gene in Poland as: 5382insC [exon 20], 4153delA [exon 11.17], and 300 T > G [exon 5]. PFS (progression free survival) and OS (overall survival) were determined by Kaplan-Meier analysis with log rank test, univariate comparisons, and multivariate regression analysis using Cox proportional hazards model.

**Results:**

Of the 125 patients, the founder mutations of *BRCA1* were reported in 17 patients (13.6 %). The median OS was longer for *BRCA* mutated patients (not reached vs 35.6 months, *p* = 0.041). PFS was similar for both kinds of ovarian cancer. In multivariate analysis, age ≥70 years, suboptimal surgery, and *BRCA1* wild type were poor prognostic factors. The *BRCA1* mutation reduced the likelihood of death in ovarian cancer by 86 % (HR 0.14; CI: 0.032-0.650, *p* = 0.012).

**Conclusion:**

In conclusion, we found better overall survival for ovarian cancer patients with *BRCA1* germline mutations in comparison with patients without these mutations (sporadic) ovarian cancer. Thus, *BRCA1* germline mutations appear to be an independent prognostic factor for ovarian cancer.

## Introduction

Ovarian cancer is the leading cause of death in women from gynecological malignant diseases. The lifetime probability of ovarian cancer in the general population is approximately 1.6 %. This risk increases to 5 % for women with one first-degree relative with ovarian cancer and to 7.2 % for women with two or three relatives with ovarian cancer [1].

*BRCA1* and *BRCA2* genes are high-penetrating with important roles during tumorigenesis. Both genes encode proteins that interact with a machinery of recombination of DNA or DNA repair pathways [2]. Twenty percent of breast cancer has familial basis and approximately 5 % to 10 % of breast cancer is hereditary. Two-thirds of these hereditary cancers occur in carriers with mutations of *BRCA1* or *BRCA2* which are germline mutations [3]. Some preclinical studies have shown that *BRCA1* appears to be an important responding factor to both DNA-damaging (platimun-compounds) and taxane-based chemotherapy [4]. Molecularly, these anticancer agents are to be as crucial modulators of *BRCA*-dependent pathways, independently of detected *BRCA1/2* mutations defined germline or sporadic [5].

The *BRCA*ness phenotype may be a result of defective homologous recombination related to several mechanisms, including epigenetic hypermethylation of the *BRCA1* promoter, somatic mutation of *BRCA1/2*, or loss of function mutations in other homologous recombination orchestrating molecules. The complex profile of the *BRCA*ness phenotype correlates with responsiveness to platinum and poly-ADP ribose polymerase (PARP) inhibitors [6]. On the other hand, there have been known at least, two molecular mechanisms responsible for chemotherapy resistance and recurrence of ovarian cancer such as 1.secondary mutations restoring *BRCA1/2*, and 2.high levels of PARP, Fanconi anemia proteins and P53 [7, 8]. Determination of the *BRCA1/2* status may be a relevant clinical biomarker both for survival prognosis and prediction both for response or resistance to chemotherapy in sporadic ovarian cancer [9, 10].

*BRCA* mutations may have impact on better survival of patients with ovarian cancer when compared with those without mutations. Several studies have investigated the possible effects of *BRCA1* mutation on clinical and pathologic characteristics defined by earlier age of onset in any mutation carriers with the possible better response to platinum-based chemotherapy, but results of these studies were inconclusive. Some of these studies have demonstrated longer survival in epithelial invasive ovarian cancer patients [11, 12] who are *BRCA1* mutation carriers in comparison with noncarriers, but other investigators did not find a survival benefit in *BRCA1* mutation carriers [13–15]. Recently reported results have revealed that after 3 years since the ovarian cancer diagnosis, the presence of a *BRCA1* or *BRCA2* mutation was associated with a clinically better prognosis (HR 0.68; 95 % CI: 0.48–0.98, *p* = 0.03), which has not been sustained 10 years after the diagnosis of ovarian cancer (HR 1.00; 95 % CI: 0.83–1.22, *p* = 0.90) [16, 17].

The aim of our study was to assess clinical features and treatment outcomes in ovarian cancer patients, having a founder mutation in *BRCA1* gene in comparison with clinical results of sporadic ovarian cancer patients.

## Patients and methods

### Patients

A consecutive series of 125 patients with ovarian cancer diagnosed and treated at the Military Institute of Medicine in Warsaw, Poland, between 2002–2008 was studied. All patients underwent surgery defined as radical, optimal tumor debulking with residual disease < 1 cm, or suboptimal as residual disease > 1 cm. The pathology reports were classified as epithelial ovarian cancer serous, endometrioid, mucinous, clear cell, mixed, or unspecified. Chemotherapy was used in all patients. Mutation analysis was performed in all enrolled patients. The study protocol was approved by the local Ethics Committee (The Resolution of The Bioethics Committee of Military Institute of Medicine at Warsaw, No 48/WIM/2008 data November, 19^th^ 2008), and written informed consent was obtained from all participants of the study.

### Chemotherapy regimen

The first line chemotherapy consisted of 6 courses. The first version of chemotherapy regimen consisted of 135 mg/m^2^ of intravenous infusion paclitaxel over 24-h on day 1 followed by 75 mg/m^2^ of intravenous infusion cisplatin on day 2. The second version of chemotherapy regimen consisted of 175 mg/m^2^ of intravenous infusion paclitaxel over 3-h on day 1 followed by AUC6 according to Calvert formula of intravenous 30-min infusion carboplatin on day 2. Standard premedication (dexamethasone 20 mg, ranitidinum 50 mg, clemastinum 2 mg) was given intravenously to prevent hypersensitivity reaction to paclitaxel. Treatments were administered every 3 weeks. The third version of chemotherapy was based upon carboplatin AUC5 according to Calvert formula of intravenous 30-min infusion. The fourth version of treatment was given as neoadjuvant triple chemotherapy regimen based upon paclitaxel (175 mg/m^2^ over 3-h intravenously), carboplatin (AUC5 intravenously), and caelyx (20 mg/m^2^ intravenously) or epirubicin (50 mg/m^2^ intravenously) which was administered for three courses before debulking surgery with further continuation of standard chemotherapy with paclitaxel and carboplatin in mentioned doses up to total number of six courses. The fifth version of chemotherapy was a regimen based upon combination of gemcitabine (1000 mg/m^2^ intravenously on days 1,8 every 21 days) and cisplatin (75 mg/m^2^ intravenously on day 1 every 21 days) given as the primary chemotherapy as well.

### Mutational analysis

High-molecular-weight DNA was isolated from peripheral blood leukocytes by nonenzymatic and rapid method described by Lahiri and Nurnberger [18]. Mutation analysis was performed for three common in Poland founder mutations in *BRCA1* (5382insC – exon 20; 4153delA – exon 11.17) by a multiplex allele-specific polymerase chain reaction (PCR) assay. 300 T > G – exon 5 mutation generates a novel restriction enzyme site. This mutation can be detected after digesting amplified DNA with Ava II. To show the different *BRCA1* alleles, the PCR products were subjected to horizontal electrophoresis in a 2.0 % agarose gel and stained with ethidium bromide. We analyzed samples of all enrolled patients [19].

### Statistical analysis

Demographic data are shown as median or mean with standard deviation (SD) and 95 % confidence interval (CI). Relationships between categorical variables were assessed using the Chi-square test, Yates-corrected Chi-square test or Mann–Whitney *U* test. Progression-free survival (PFS) was calculated from the start date of chemotherapy to the first evidence of treatment failure. Overall survival (OS) was defined as the time interval between the date of starting chemotherapy to death of any cause. PFS and OS were estimated using the Kaplan-Meier methods and differences in survival were compared by using log-rank test. On univariate comparisons of survival between groups, statistical significance was assessed using the Cox-Mantel test. Multivariate regression analyses were performed using the Cox proportional hazards model. A *p* value of <0.05 was considered statistically significant. For calculations we used STATISTICA for Windows Version 7.0 software.

## Results

### Patient characteristics

The study population involved a series of 125 consecutive patients with ovarian cancer treated primary surgically and further chemotherapeutically. Table 1 shows patient characteristics. The patients’ age ranged from 28 to 85 years (median, 55 years). Most patients were in advanced stage ovarian cancer (III and IV stages, 101/125: 80.8 %). More than half of patients were diagnosed pathologically with serous ovarian cancer. Standard chemotherapy was given to participated patients in this study with exception of one patient received gemcitabine combined with cisplatin as the primary chemotherapy.

**Table 1:** Patient characteristics

Number of patients	*n* = 125
Median (range) age at diagnosis in years	55 (28–85)
Performance status WHO:	
▪ 0	15.2 % (19/125)
▪ 1	77.6 % (97/125)
▪ 2	7.2 % (9/125)
BSA (m^2^)	1.69 (95 % CI; 1.65–1.72)
FIGO stage:	
▪ I	11.2 % (14/125)
▪ II	8.0 % (10/125)
▪ IIIA	5.6 % (7/125)
▪ IIIB	7.2 % (9/125)
▪ IIIC	56.0 % (70/125)
▪ IV	12.0 % (15/125)
Chemotherapy regimen:	
▪ Paclitaxel/cisplatin	43.2 % (54/125)
▪ Paclitaxel/carboplatin/ caelyx	8.8 % (11/125)
▪ Paclitaxel/carboplatin	36.8 % (46/125)
▪ Carboplatin	8.8 % (11/125)
▪ Paclitaxel/carboplatin/epirubicin	1.6 % (2/125)
▪ Gemcitabine/ cisplatin	0.8 % (1/125)
Type of histology:	
▪ Serous	54.4 % (68/125)
▪ Endometrioid	26.4 %(33/125)
▪ Mucinous	7.2 % (9/125)
▪ Clear cell	4.0 % (5/125)
▪ Mixed	2.4 % (3/125)
▪ Unspecified	5.6 % (7/125)
Grading:	
▪ 1	3.2 % (4/125)
▪ 2	32.8 % (41/125)
▪ 3	23.2 % (29/125)
▪ Unspecified	40.8 % (51/125)
Primary surgery:	
▪ Radical	19.2 % (24/125)
▪ Optimal (<1 cm)	42.4 % (53/125)
▪ Suboptimal (>1 cm)	38.4 % (48/125)

Three founder mutations (5382insC – exon 20; 4153delA – exon 11.17; 300 T > G – exon 5) dominating in Poland were tested. Founder mutations of the *BRCA1* gene were noted with the rate of 13.6 %. The most frequent (9.6 %) mutation was in exon 20 (5382insC). Table 2 shows the comparative distribution of founder mutations of *BRCA1* in ovarian cancer patients.

**Table 2:** Distribution of founder mutations of the *BRCA1* gene in ovarian cancer patient

Germline mutation	All ovarian cancer (*n* = 125)
5382insC [exon 20]	12/125 (9.6 %)
300 T > G [exon 5]	3/125 (2.4 %)
4153delA [exon 11.17]	2/125 (1.6 %)
All mutations	17/125 (13.6 %)

Table 3 presents clinical and pathological ovarian cancer characteristics with the *BRCA1* gene status. Younger patients with ovarian cancer were seen among *BRCA1* mutation carriers in comparison with noncarriers (median age 47 vs 56, respectively, *p* = 0.004).

**Table 3:** Clinical and pathological ovarian cancer characteristics determined by the *BRCA1* gene status

Status *BRCA1*	Wild type (*n* = 108)	Germline mutation (*n* = 17)	Statistics	*p* value
Median (range) age at diagnosis in years	56 (28–85)	47 (39–70)	−2.859^a^	***0.004***
Performance status WHO:				
▪ 0	15	4		
▪ Other	93	13	0.44^b^	0.51
FIGO stage:				
▪ Early (I, II)	22	3		
▪ Advanced (III,IV)	86	14	0.07^b^	0.95
Chemotherapy regimen:				
▪ With paclitaxel	97	16		
▪ Without paclitaxel	11	1	0.01^b^	0.91
Chemotherapy regimen:				
▪ With cisplatin	47	8		
▪ With carboplatin	61	9	0.1^c^	0.75
Type of histology:				
▪ Serous	57	11		
▪ Other	51	6	0.84^c^	0.36
Grading:				
▪ 1 and 2	38	7		
▪ 3 and unspecified	70	10	0.23^c^	0.63
Primary surgery:				
▪ Optimal (<1 cm)	68	9		
▪ Suboptimal (>1 cm)	40	8	0.62^c^	0.43

^a^Mann–Whitney *U* test

^b^Yates-corrected Chi-square test

^c^Chi-square test

### Progression free survival analysis incorporated the status of the *BRCA1* gene and other clinical variables

On univariate analysis for PFS, patients with advanced stage, serous histology, nonoptimal debulking surgery with residual tumor > 1 cm, and without paclitaxel in the chemotherapy regimen correlated with worse PFS. *BRCA1* gene status was not a significant factor for PFS. Detailed results of univariate and multivariate analyses are shown in Table 4. The FIGO stage, serous histology, optimal debulking surgery, and chemotherapy with paclitaxel combined with platinum compound were significant prognostic factors for PFS. The status of *BRCA1* was not of clinical value as prognostic factor after using multivariate analysis for PFS.

**Table 4:** Univariate and multivariate analyses for PFS of ovarian cancer

Univariate analysis			
Variable	n (%)	Median (months)	*p* value
Age:			
<70	118 (94.4 %)	10.4	0.51
≥70	7 (5.6 %)	16.0	
Performance status WHO:			
▪ 0	19 (15.2 %)	14.7	0.06
▪ Other	106 (84.8)	21.4	
FIGO stage:			
▪ Early (I,II)	24 (19.2 %)	NR	**<** ***0.001***
▪ Advanced (III, IV)	101 (80.8 %)	13.4	
Chemotherapy regimen:			
▪ With paclitaxel	113 (90.4 %)	16.8	***0.032***
▪ Without paclitaxel	12 (9.6 %)	10.3	
Chemotherapy regimen:			
▪ With cisplatin	55 (44.0 %)	23.0	0.24
▪ With carboplatin	70 (56.0 %)	13.5	
Type of histology:			
▪ Serous	68 (54.4 %)	13.2	**<** ***0.001***
▪ Other	57 (45.6 %)	34.8	
Grading:			
▪ 1 and 2	45 (36.0 %)	16.0	0.40
▪ 3 and unspecified	80 (64.0 %)	14.6	
Primary surgery:			
▪ Optimal (<1 cm)	77 (61.6 %)	24.7	**<** ***0.001***
▪ Suboptimal (>1 cm)	48 (38.4 %)	11.0	
*BRCA1* status:			
▪ Wild type	108 (86.4 %)	14.6	0.58
▪ Germline mutation	17 (13.6 %)	21.5	
Multivariate analysis			
Variable	HR (95 % CI)		*p* value
FIGO stage:			
▪ Early (I,II)	0.21 (0.071 – 0.634)		***0.006***
▪ Advanced (III, IV)			
Chemotherapy regimen:			
▪ With paclitaxel	0.46 (0.242 – 1.020)		***0.034***
▪ Without paclitaxel			
Type of histology:			
▪ Serous	0.55 (0.318 – 0.916)		***0.027***
▪ Other			
Primary surgery:			
▪ Optimal (<1 cm)	0.54 (0.308 – 0.804)		***0.011***
▪ Suboptimal (>1 cm)			
*BRCA1* status:	NG		NS
▪ Wild type			
▪ Germline mutation			

*NG* not given, *NR* not reached, *NS* statistically not significant

### Overall survival analysis incorporated the status of the *BRCA1* gene and other clinical variables

**Fig. 1:**
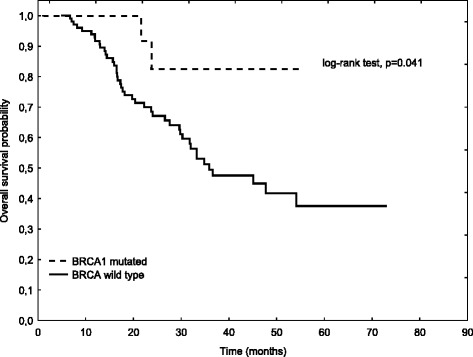
Overall survival stratified by *BRCA1* status

On univariate analysis for OS (Table 5), we show that age at ovarian cancer onset ≥70 years, advanced stage, serous histology, suboptimal surgery, and use of chemotherapy regimen without paclitaxel are related to shorter OS. Interestingly, *BRCA1* status appeared to be of clinical value as a prognostic factor for OS in ovarian cancer patients. Carriers of any mutation of the *BRCA1* gene with ovarian cancer had better survival at two years in comparison with noncarriers. Figure 1 presents OS in patients with ovarian cancer stratified by *BRCA1* status with median not reached in carriers of *BRCA1* mutations vs median of 35.6 months for noncarriers (*p* = 0.041). In multivariate analysis (Table 5), age ≥ 70 years, suboptimal surgery and expression of wild type of the *BRCA1* gene correlate with poor prognosis for OS. *BRCA1* germline mutations reduced by 86 % likelihood of death in our ovarian cancer patients (HR 0.14 [CI: 0.032–0.650], *p* = 0.012).

**Table 5:** Univariate and multivariate analyses for OS of ovarian cancer

Variable	n (%)	Median (months)	*p* value
Age:			
<70	118 (94.4 %)	46.0	***0.022***
≥70	7 (5.6 %)	14.8	
Performance status WHO:			
▪ 0	19 (15.2 %)	NR	0.28
▪ Other	106 (84.8)	36.2	
FIGO stage:			
▪ Early (I,II)	24 (19.2 %)	NR	***0.003***
▪ Advanced (III, IV)	101 (80.8 %)	33.3	
Chemotherapy regimen:			
▪ With paclitaxel	113 (90.4 %)	46.1	***0.035***
▪ Without paclitaxel	12 (9.6 %)	16.6	
Chemotherapy regimen:			
▪ With cisplatin	55 (44.0 %)	50.5	0.24
▪ With carboplatin	70 (56.0 %)	32.3	
Type of histology:			
▪ Serous	68 (54.4 %)	33.2	***0.047***
▪ Other	57 (45.6 %)	NR	
Grading:			
▪ 1 and 2	45 (36.0 %)	49.8	0.53
▪ 3 and unspecified	80 (64.0 %)	35.6	
Primary surgery:			
▪ Optimal (<1 cm)	77 (61.6 %)	51.8	***0.028***
▪ Suboptimal (>1 cm)	48 (38.4 %)	30.0	
*BRCA1* status:			
▪ Wild type	108 (86.4 %)	35.6	***0.041***
▪ Germline mutation	17 (13.6 %)	NR	
Multivariate analysis			
Variable	HR (95 % CI)		*p* value
Age:			
<70	0.15 (0.053 – 0.417)		**<** ***0.001***
≥70			
Primary surgery:			
▪ Optimal (<1 cm)	0.39 (0.205 – 0.715)		***0.003***
▪ Suboptimal (>1 cm)			
*BRCA1* status:			
▪ Germline mutation	0.14 (0.032 – 0.650)		***0.012***
▪ Wild type			

*NR* not reached

## Discussion

Our results found the total rate of *BRCA1* mutations was 13.6 % in 125 ovarian cancer patients. This result is in accordance with other previously published studies in the Polish population with germline mutations in *BRCA1* and *BRCA2* in 13.5 % and 13.9 % of patients, respectively [20, 21]. We also show that *BRCA1* germline mutations are associated with good prognosis for patients with ovarian cancer and OS was better for carriers of *BRCA1* mutations (HR 0.14, 95 % CI: 0.032–0.650, *p* = 0.012) than for noncarriers. This observation is in accordance with results of other authors, for example, Rubin et al. [22] for the first time determined that ovarian cancer associated with *BRCA1* mutations had a more favorable clinical course, and it was confirmed by other studies in which this advantage in survival of mutation carriers was reported to be an independent protective factor [23].

Here we report that PFS assessed both on univariate and on multivariate analyses (*BRCA1* mutations vs *BRCA1* wild type, median 21.5 months vs 14.6 months, *p* = 0.58, respectively) show a slight favor with predictive value for patients with mutated *BRCA1* but without statistical significance. We present that FIGO stage, serous histologic type, primary debulking surgery and chemotherapy based upon combination of platinum compound with paclitaxel, are typical clinical variables that correlate with PFS. Among 71 Jewish patients with ovarian cancer, 22 had *BRCA1* three germline mutations and one truncating mutation, and 12 had *BRCA2* truncating mutations vs 37 patients with sporadic ovarian cancer. Overall survival was improved in patients with mutated forms of *BRCA* in comparison with sporadic ovarian cancer patients (91 months vs 54 months, respectively; *p* = 0.046). In accordance with our results, the authors showed that patients with *BRCA* mutations had slightly longer disease free survival but not statistically significant (49 months vs 19 months, respectively; *p* = 0.16) [24]. Tan et al. [25] coined the term of a clinical syndrome of *BRCAness* which relies on better prognosis for response rate after first- line chemotherapy and at subsequent recurrences, overall survival, serous histology (but not necessary), and TFI (treatment-free interval defined as the time between each line of treatment calculated from the date of the last course of the previous chemotherapy to the date of the first cycle of the next chemotherapy). In ovarian cancer patients with *BRCA* mutations the median TFI for three lines of chemotherapy was significantly longer than in sporadic ovarian cancer patients (first-line, *p* < 0.001; second-line, *p* < 0.015; third-line, *p* = 0.002, respectively). The median overall survival for *BRCA*-positive ovarian cancer patients was better than in controls (from the time of diagnosis 8.4 years vs 2.9 years, respectively; *p* < 0.002) [25, 26]. Gallagher et al. [27] reported in a group of 110 patients (36 with three germline mutations of *BRCA* genes (20 *BRCA1* mutations, 16 *BRCA2* mutations) vs 74 controls) that ovarian cancer patients with *BRCA* mutations had better survival (median not reached vs 67.8 months for controls, respectively; *p* = 0.02). The multivariate analysis sustained crucial role of *BRCA* mutations in overall survival prognosis (HR 0.36; 95 % CI 0.14–0.93) but disease-free survival was not significantly different between *BRCA* mutations and control ovarian cancer patients (median 26.9 months vs 24.0 months, respectively; *p* = 0.30).

In our study, the age at the onset of ovarian cancer for *BRCA1* wild type was almost 10 years postponed than in carriers of *BRCA*1 mutations (median 56 years vs 47 years, respectively, *p* = 0.004). Thigpen et al. [28] decided to determine major prognostic factors in 2123 ovarian cancer patients who were studied in the six GOG (Gynecologic Oncology Group) clinical trials. It turned out that only three factors had impact on prognosis for overall survival of the entire investigated population, i.e., age, volume of residual disease, and performance status. Based upon the results of studied patients, elder patients with ovarian cancer (>69 years) had poorer survival. Therefore, age seems to have indirect roles in better survival of patients with mutated *BRCA1* through better response to chemotherapy, better tolerance of such a therapy without other health problems which typically are associated with elder patients. But on the other hand, the second-line chemotherapy due to relapsed ovarian cancer could be used in elder patients after detailed assessment of performance status rather than making-decision based primarily on the age [29].

Malignant diseases are the final results of incorrect interactions between immune-surveillance in a healthy organism and unrestricted proliferation of a small portion of cells that constitute a solid tumor or leukemia. The fundamental process that is primarily responsible for tumorigenesis embraces changes in the DNA sequences of the genomes of malignant cells. Recently accumulating knowledge clearly shows that detection cancer genome structural changes at the levels of DNA sequences (mainly somatic mutations) and epigenetic alterations lead to improper functions in cells that constantly become malignant. This dynamic process defines cancer as an evolutionary entity determining highly probabilistic events providing finally advanced malignant disease [30]. Naturally occurring during malignant progression, genome changes and epigenetic alterations seem to have impact on detection of molecular abnormalities with clinical usefulness at a bed in oncology ward. TCGA (The Cancer Genome Atlas) project is a well-know initiative to establish a series of somatic mutations and epigenetic alterations in human cancer samples in relation to clinical data. Analyses performed in 489 ovarian cancer samples (mainly high-grade serous ovarian adenocarcinoma) revealed changes in expression of mRNA, noncoding RNA, methylation of promoter of many genes, and changed DNA copy numbers. The most important gene mutations were ascribed to P53 detected in 96 % of investigated samples. Other low prevalent with statistical relevance aberrations in the form of somatic mutations were noted in such genes as *NF1*, *BRCA1*, *BRCA2*, *RB1* and *CDK12*. In 168 genes, the authors detected promoter methylation alterations and prognosis for survival was performed in patients with *BRCA* mutations and *CCNE1* aberrations [31].

Based on a cohort of 316 ovarian cancer patients taken from TCGA, 77.8 % had *BRCA1/2* wild type, 8.5 % had germline mutations, 3.1 % had somatic mutations, and 10.4 % had *BRCA1* hypermethylation. Detailed analyses showed that the only prognostic factor for OS in ovarian cancer was mutated *BRCA2* (HR 0.33; 95 % CI: 0.16–0.69, *p* = 0.003). Taking into account platinum-based chemotherapy efficacy, mutated *BRCA2* gene had significantly longer PFS (HR 0.40; 95 % CI: 0.22–0.74, *p* = 0.004) [32]. Also, Hyman et al. [33] reported that better survival was seen in patients carrying *BRCA2* mutations [HR 0.20; 95 % CI 0.06–0.65, *p* = 0.007] compared with either *BRCA1* carriers [HR 0.70; 95 % CI 0.36–1.38, *p* = 0.31] or noncarriers. The presented results are surprising, especially in the context of many published studies showing prognostic role of germline mutations of *BRCA1* gene. It seems that patient selection through a specific definition of primary response to adjuvant chemotherapy or variety in stage distribution will play a crucial role in the process of analysis and interpretation of collected results, but further studies should be planned as randomized and controlled with statistically well-founded number of participating patients. Some commentaries on the potential role of *BRCA2* mutations as a real prognostic factor in ovarian cancer patients show such a particular role of patient selection and differences in surgical and oncologic managements [34].

## Conclusions

To summarise, our findings confirm previous reports that improved overall survival associated with *BRCA1* mutations carried in ovarian cancer patients. Both univariate and multivariate analyses show that the *BRCA* gene status dychotomizes ovarian cancer patients to better and worse prognosis for overall survival (*BRCA1* germline mutations constitute better prognosis than *BRCA1* wild type). The most important limitation of our study is a small number of patients (125 patients, 17 with germline mutations of *BRCA1* and 108 controls). Moreover, we focused only on three *BRCA1* germline mutations typical for the Polish population that reflects low frequency such patients among consecutive patients visiting an oncologist. However, our results are consistent with other reports discussed above.

### Ethical statement

The study was approved by the local Ethics Committee Military Institute of Medicine, Warsaw, Poland.
